# VitiForge: a new procedural pipeline approach for grapevine disease identification under data scarcity

**DOI:** 10.3389/fpls.2025.1706973

**Published:** 2025-11-14

**Authors:** Eduardo Farinati Leite, Lucas Brizola Fontoura, Augusto de Freitas, Giusepe Disconzi Dallegrave, Rafael Volpe de Freitas, Vinícius Silveira Mello, João Francisco Valiati

**Affiliations:** 1 Center for Embedded Devices and Research in Digital Agriculture (CEDRA) of SENAI-RS, São Leopoldo, Brazil; 2 SENAI Innovation Institute for Sensing Systems (ISI-SIM), São Leopoldo, Brazil

**Keywords:** deep learning, plant disease recognition, convolutional neural network, synthetic data, precision agriculture

## Abstract

Early identification of grapevine diseases is critical for reducing yield losses and ensuring sustainable viticulture. CNNs trained on benchmark datasets such as PlantVillage often achieve near-perfect accuracy, yet this performance fails to translate to real-world field conditions where lighting, backgrounds, and lesion appearance vary widely. To address challenges of data scarcity and imbalance, this study introduces VitiForge, a novel procedural synthetic imagery pipeline for generating realistic synthetic grape leaf textures representing healthy, Black Rot, Esca, and Leaf Blight conditions. VitiForge is systematically evaluated against GAN-based augmentation through a data ablation study on PlantVillage and FieldVitis, a curated field dataset, using MobileNetV2, InceptionV3, and ResNet50V2 classifiers. Results show that VitiForge significantly improves performance in low-data regimes, enabling model training even without real samples, whereas GAN augmentation proves more effective once sufficient real data is available. On field imagery, VitiForge often matched or surpassed GAN-based methods, particularly when paired with MobileNetV2. These findings highlight the complementary roles of procedural and GAN-based synthetic data: VitiForge offers flexibility and scalability under cross-domain and data-scarce conditions, while GANs enhance realism and variability when ample data exists. Together, they support the development of robust and generalizable models for automated grape disease detection in precision agriculture.

## 1 Introduction

Early detection of plant diseases, often manifested as visible patterns on leaves, is critical for minimizing crop yield losses. Globally, plant diseases account for more than 30% of annual crop losses, equating to hundreds of billions of dollars in damage (Gai and Wang, 2024). Traditional monitoring methods are labor-intensive, time-consuming, and reliant on expert knowledge, underscoring the need for automated solutions. Viticulture exemplifies the opportunities in this field. Grapes represent an important fruit crop with significant global economic importance (Li et al., 2023); however, grapevine diseases such as Black Rot, Leaf Blight, Downy Mildew and Esca pose substantial threats to cultivation, motivating intensive research into automated disease detection approaches (Xie et al., 2020; Tang et al., 2020; Lu X. et al., 2022; Zhu et al., 2021).

Among these diseases, Black Rot (*Guignardia bidwellii*) and Esca are particularly destructive, experiencing up to 100% yield loss from Black Rot (Szabó et al., 2023) with heavily infected vineyards.

Recent advances in Artificial Intelligence (AI) and image processing have enabled more precise plant disease detection, where automated workflows typically involve steps such as image acquisition, segmentation, feature extraction, and lesion classification. Within this framework, deep learning models have shown strong effectiveness in recognizing disease symptoms in the visible spectrum (Upadhyay et al., 2025), with a wide range of segmentation and feature extraction strategies also explored to improve performance (Khirade and Patil, 2015; Székely et al., 2024).

Convolutional Neural Network (CNN)-based models, such as MobileNet, Inception, and ResNet, have shown great promise for grape disease classification and have achieved impressive accuracy on benchmark datasets like PlantVillage (Mohanty et al., 2016), with some models reaching near-perfect results under controlled conditions (Kunduracioglu and Pacal, 2024; Lu X. et al., 2022). However, their performance often degrades sharply when applied to real-world images, where uneven lighting, complex backgrounds, and subtle lesion patterns introduce significant challenges (Barbedo, 2022). Transfer learning can alleviate some of these limitations (Morellos et al., 2022), but the domain gap remains a persistent obstacle.

Despite their success, CNN models rely on large, representative, and balanced datasets to achieve robust performance across diverse conditions (Dablain et al., 2023; Albattah and Khan, 2025). In the context of plant pathology, however, constructing such datasets is particularly challenging. Plant diseases are inherently variable, influenced by environmental factors, growth stages, and pathogen interactions, which makes capturing sufficient examples of all relevant symptoms extremely difficult. Furthermore, disease occurrence is region-dependent and seasonal, meaning that data collection requires significant time and geographic coverage. As a result, available datasets are often limited, imbalanced, and narrowly focused (Pacal et al., 2024).

Synthetic data has emerged as a promising solution to this challenge. Several studies have shown that synthetic augmentation can substantially reduce the reliance on large real-world datasets (Nowruzi et al., 2019), and agricultural applications have already employed synthetic data effectively in tasks such as crop phenotyping (Toda et al., 2020). In the context of plant disease detection, Generative Adversarial Networks (GANs) have become a widely used approach for generating synthetic plant images, particularly to expand minority classes and balance datasets, thereby improving the robustness of training (Xie et al., 2020; Tang et al., 2020; Liu et al., 2020; Jin et al., 2022).

Taking an alternative approach, this work proposes VitiForge, a novel scalable pipeline for generating realistic synthetic grape leaf images using a procedural methodology. The approach produces diverse, customizable datasets that realistically represent grape leaves and disease patterns under varied conditions. The effectiveness of procedurally generated synthetic grape leaf images is evaluated as a data augmentation strategy for classical AI models in disease detection. Benchmarking is conducted through a data ablation strategy applied to splits of the PlantVillage dataset and FieldVitis, a curated dataset of field images assembled from public sources, comparing three approaches: (i) real data only, (ii) real data with GAN based augmentation, and (iii) real data with VitiForge augmentation. The main contributions of this work can be summarized as follows:

1. The development of VitiForge, a scalable framework for synthesizing realistic grapevine leaf imagery by procedurally modeling disease patterns (Black Rot, Esca, and Leaf Blight) and environmental variability such as lighting, orientation, and background clutter.
2. The introduction of FieldVitis, a curated dataset of grapevine leaves collected from multiple public sources to reflect the real-world variability of vineyard imagery, providing a valuable benchmark for evaluating model generalization under realistic field conditions. It is available in Zenodo at https://doi.org/10.5281/zenodo.17307846.
3. A comprehensive data ablation study on PlantVillage and FieldVitis, demonstrating that VitiForge consistently improves performance in low-data scenarios and even enables model training in complete absence of real samples, establishing its viability as a zero-data solution.
4. A comparative analysis across three CNN architectures (MobileNetV2, InceptionV3, and ResNet50V2), highlighting architecture-specific responses to augmentation strategies, with MobileNetV2 achieving the strongest and most consistent gains.

## 2 Related work

This research on automated grape disease detection intersects with three main areas: deep learning approaches for plant pathology, synthetic data augmentation in agriculture, and procedural image generation for computer vision.

### 2.1 Deep learning for grape leaf disease identification

Deep learning techniques have been widely applied to grape disease classification, with most early work demonstrating strong results on controlled datasets such as the PlantVillage dataset (Mohanty et al., 2016), which comprises 54,306 images of diseased and healthy leaves across 14 crop species and 26 diseases, including approximately 4,000 grape leaf images. Using this resource, Geetharamani and Pandian (2019) developed a nine-layer CNN that achieved accuracies above than 90%. Similarly, Tang et al. (2020) proposed a lightweight model combining ShuffleNet with squeeze-and-excitation blocks, reaching 99.14% test accuracy on PlantVillage while maintaining a compact size suitable for embedded deployment. Extensions to real-time detection have also been pursued, as in Xie et al. (2020), who introduced Faster DR-IACNN trained on an augmented PlantVillage dataset of 62,286 images, achieving 81.1% mean Average Precision (mAP).

Beyond classical CNNs, recent work has explored more sophisticated architectures and feature fusion strategies. Transformer-based designs such as Swin Transformer have demonstrated near-perfect performance, with some configurations reaching 100% accuracy on grape leaf classification (Kunduracioglu and Pacal, 2024). Hybrid models combining CNN and Transformer components have also been proposed, including Group Shuffle Residual DeformNet with Swin Transformer (Karthik et al., 2024b) and Inception ResNet with Shuffle-Transformer fusion (Karthik et al., 2024a), both reporting high classification accuracy. Other studies have investigated variations in CNN backbones, such as VGG12 with wide convolution layers (Thomkaew, 2025), deep learning approaches for multiclass grape disease classification (Fraiwan et al., 2022), and broader comparative analyses across multiple architectures (Mangaoang, 2025).

A recurring challenge emerges when models trained on controlled data are evaluated on field imagery. As emphasized by George et al. (2025), leaf disease datasets in general can be broadly divided into laboratory collections, characterized by controlled lighting, uniform backgrounds, and isolated leaf presentation, and real-world collections, which include varied environmental conditions such as wind, uneven illumination, diverse backgrounds, and occlusions from surrounding foliage. This distinction is evident in grapevine disease detection as well: Morellos et al. (2022) reported that fine-tuned CNNs achieved 100% validation accuracy on PlantVillage (a typical laboratory dataset) but dropped to 66.7% with AlexNet when tested on vineyard-collected images. Similarly, Zhu et al. (2021) observed that Black Rot detection achieved 95.79% precision and 94.52% recall on controlled indoor test images, compared to 86.69% precision and 82.27% recall on orchard images, with performance partially recovering when restricted to simple backgrounds. More complex acquisition setups such as UAV imagery have also been investigated: Li et al. (2023) introduced a multistage pipeline combining Multifusion U-Net with modified VGG-19, reaching 71.91% average segmentation accuracy under challenging conditions with low light and motion interference.

To reduce this domain gap, several field-oriented datasets have been developed, like observed in (Alessandrini et al., 2021) (Dharrao et al., 2025), and (Lu X. et al., 2022) that intended to expand coverage to field conditions, yet their size, class balance and diversity remain limiting factors in building broadly generalizable models.

### 2.2 Synthetic data augmentation in agriculture

To address the limitations of dataset size, class balance, and diversity, data augmentation is a standard strategy in plant disease detection pipelines. As noted by George et al. (2025), traditional augmentation approaches can be broadly divided into position-based (mirroring, clipping, rotation) and color-based (brightness, contrast, saturation) transformations, with more advanced techniques such as CutMix and MixUp combining sample images through linear interpolation or region-level patching. In addition, masking-based background replacement has been proposed to better mimic real-world conditions (Benabbas et al., 2024). While these methods improve generalization, they are limited in their ability to generate genuinely new samples or replicate the variability of field conditions.

Beyond these traditional approaches, GANs have been widely investigated for synthetic augmentation in agricultural computer vision. Reviews by Sampath et al. (2021) and Lu Y. et al. (2022) document their effectiveness for addressing dataset imbalance and scarcity, particularly when constrained by seasonal or geographic limitations. GANs have been applied across diverse crops: Giuffrida et al. (2017) pioneered a DCGAN-style framework for synthesizing Arabidopsis images, Zhao et al. (2022) used GANs for wheat disease generation, Li et al. (2024) developed SugarcaneGAN for feature expansion in sugarcane diseases, and Ramadan et al. (2024) demonstrated synthetic augmentation for wheat disease classification. Other work includes Kierdorf et al. (2022), who applied conditional GANs to estimate occluded grapevine berry counts by generating synthetic leaf–berry compositions, and Barth et al. (2020), who employed CycleGAN to enhance realism in plant images for part segmentation tasks, Cap et al. (2022) introduced LeafGAN, a model that leverages segmentation masks on top of CycleGAN to generate cucumber disease-specific imagery.

In grapevine pathology, specialized GAN architectures have been developed to capture the unique features of grape leaf diseases. Liu et al. (2020) introduced Leaf GAN, not to be confused with LeafGAN, which incorporated degressive-channel deconvolutions in the generator and dense connectivity with instance normalization in the discriminator, stabilized with a deep regret gradient penalty. Trained on 4,062 PlantVillage grape leaf images, Leaf GAN synthesized 8,124 additional samples with enhanced lesion visibility and achieved lower Fréchet Inception Distance (FID) than DCGAN and WGAN. Incorporating these GAN-generated samples improved downstream CNN classifiers, with Xception reaching 98.7% accuracy.

Building on this direction, Jin et al. (2022) developed GrapeGAN, an unsupervised GAN architecture for grape leaf disease image enhancement. Its U-Net-like generator with reorg downsampling, residual blocks, and feature concatenation preserved fine lesion textures, while a discriminator combining convolutional blocks with a capsule network enforced structural integrity and reduced artifacts such as petiole misalignment.

Taking an alternative approach, Zhang et al. (2021) employed a multi-feature fusion Faster R-CNN (MF^3^ R-CNN) for soybean leaf disease detection under field conditions, with synthetic data augmentation through compositing diseased leaves from scene captures into real soybean field backgrounds, followed by reflection, rotation, and color perturbation. Notably, trained solely on synthetic data, MF^3^ R-CNN was able to generalize to real soybean field imagery, robustly identifying virus disease, frogeye leaf spot, and bacterial spot despite occlusion and background clutter.

Recent developments have explored alternative generative approaches in agricultural contexts. Muhammad et al. (2023) and Egusquiza et al. (2025) investigated the application of diffusion models for plant disease image augmentation, examining their potential for generating synthetic plant disease imagery. Heschl et al. (2025) introduced SynthSet, a methodology utilizing Denoising Diffusion Probabilistic Models and GANs for generating synthetic annotated agricultural data, with demonstrated efficacy in wheat head segmentation applications.

### 2.3 Procedural synthetic image generation for AI augmentation

Procedural image generation has been widely explored in computer vision applications where annotated data is costly or impractical to obtain. Park et al. (2021) demonstrated its effectiveness for food instance segmentation, using Blender-generated datasets to train Mask R-CNN models that performed strongly on real meal images without requiring manual annotations. Similarly, Mousavi et al. (2020) introduced a platform built on Unreal Engine for controlled synthetic dataset generation, enabling systematic variation of factors such as lighting and texture fidelity while preserving scene geometry.

In agriculture and plant-related computer vision, procedural synthesis has also shown promise. Toda et al. (2020) generated seed images across multiple crops using domain randomization, training networks that achieved 96% recall and 95% average precision when evaluated on real phenotyping tasks. Barth et al. (2018) developed a 3D modeling pipeline for *Capsicum annuum* leaves based on empirical measurements, achieving segmentation performance comparable to models trained on real annotated data while reducing annotation costs. More recently, Giakoumoglou et al. (2023) combined procedural generation with diffusion models in the Generate-Paste-Blend-Detect framework for pest monitoring, reporting competitive whitefly detection performance without large manually labeled datasets.

These examples demonstrate that procedural methods can reduce reliance on annotated data and provide explicit control over variability. However, despite their adoption in related agricultural contexts, procedural generation remains relatively unexplored in plant disease detection tasks.

## 3 Materials and methods

To evaluate the effectiveness of VitiForge for grapevine disease identification, a methodology centered on the creation, augmentation, and testing of grape leaf datasets was designed. The methods described below cover the synthetic generation pipeline, disease pattern modeling, dataset construction, ablation study design, and classifier training protocols, providing a reproducible framework for assessing performance across both laboratory and field conditions.

### 3.1 Synthetic grape leaf generation methodology

This work investigates and compares synthetic data generation as a means to improve grape leaf disease identification, focusing on four classes derived from the PlantVillage dataset: healthy leaves, and leaves affected by Black Rot, Esca, and Leaf Blight. To address different scenarios of data availability and modeling requirements, two complementary generation strategies were employed: VitiForge, a procedural rendering framework developed in this study, and a GAN-based generative framework adapted from prior work.

The procedural approach generates samples by projecting high-resolution textures onto 3D meshes and procedurally applying disease-specific patterns that replicate lesion color, shape, and distribution. Parameters such illumination, surface variation, and lesion progression can be systematically adjusted, enabling scalable and controllable dataset creation. In contrast, the GAN-based approach employs adversarial training to learn a transformation from healthy leaves to diseased variants, relying on unsupervised image-to-image translation. The second approach serves as a baseline against which the procedural method can be compared.

#### 3.1.1 VitiForge leaf generation

VitiForge’s methodology for generating synthetic imagery is grounded in the projection of real-world photographs onto 3D planes assigned with Physically Based Rendering (PBR) materials (Pharr et al., 2023). Formally, a PBR material  T on a surface S can be defined as a tuple of spatially varying fields


$$T=(\text{a}:S\to {\left[0,1\right]}^{3},\alpha :S\to [0,1],{\text{n}}_{t}:S\to S^{2},\rho :S\to [0,1])$$

where a is the albedo map (base color), α the alpha mask (opacity), ${\text{n}}_{t}$ the normal map (in tangent space), and ρ the roughness map (controlling microfacet variance).

**Figure 1:**
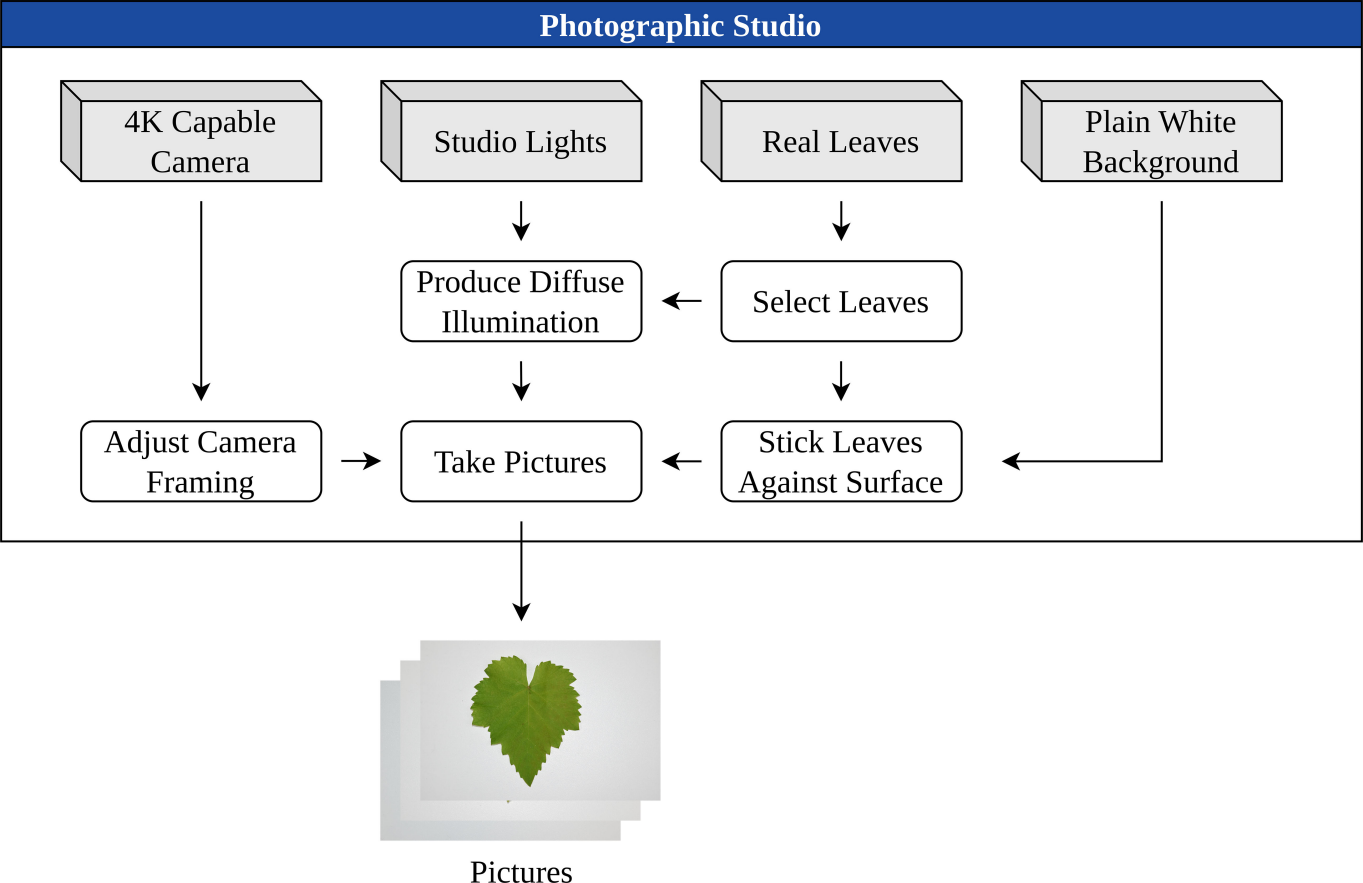
Schematic representation of the images collection pipeline.

The albedo map a was derived from high-resolution photographs of grape leaves captured against a plain white background under diffuse illumination, thereby minimizing cast shadows and enhancing surface detail. Images were subsequently processed for color correction, alignment, and optimization to ensure their suitability as texture assets. The image collection setup is illustrated in **Figure 1**.

**Figure 2:**
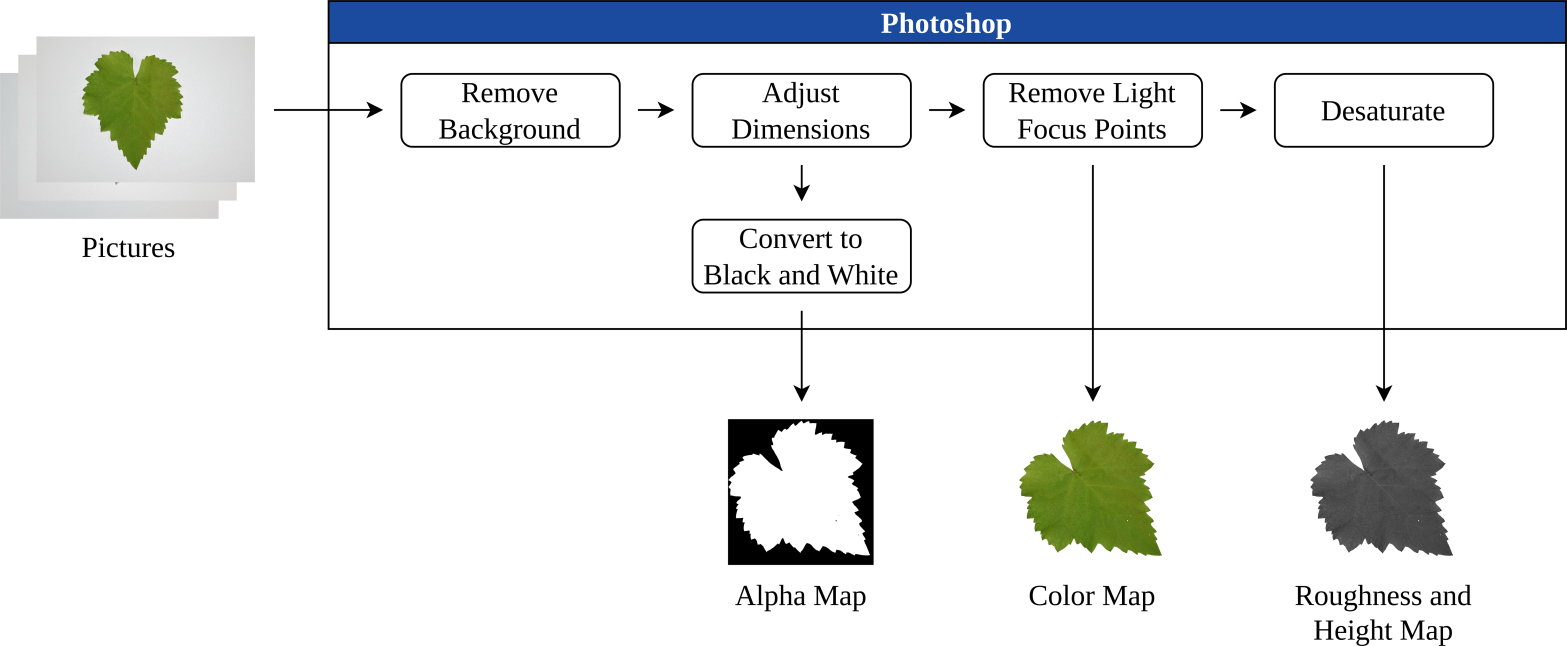
The image processing workflow.

The alpha mask *α* was generated to isolate the leaf from its background and enabling its integration into synthetic scenes. This allows systematic variation of the background, illumination, and other environmental factors. The post-processing workflow is presented in **Figure 2**.

To enhance realism, the normal ${\text{n}}_{t}$ and roughness *ρ* maps were incorporated, modulating light-surface interaction to simulate depth, venation, and subtle irregularities (Lin et al., 2014). These maps were generated from leaf photographs by creating grayscale height maps in Adobe Photoshop 2025, which were then imported into Blender 4.3.2 and applied as displacement masks. This procedure allowed the mesh to approximate real-world surface topography such as veins and roughness.

The high-polygon leaf model was subsequently paired with a simplified low-poly mesh using the caging technique (Keleşoğlu and Özer, 2021). In practice, the cage is a slightly expanded version of the low-poly geometry that fully encloses the high-poly model. During the baking process, where fine surface details are encoded into texture maps, the software casts rays from the surface of the low-poly model and uses the cage to guide their direction. This ensures that intricate details from the high-poly surface, such as grooves, ridges, and venation, are accurately projected into *a*, while avoiding artifacts due to misaligned ray projections.

**Figure 3:**
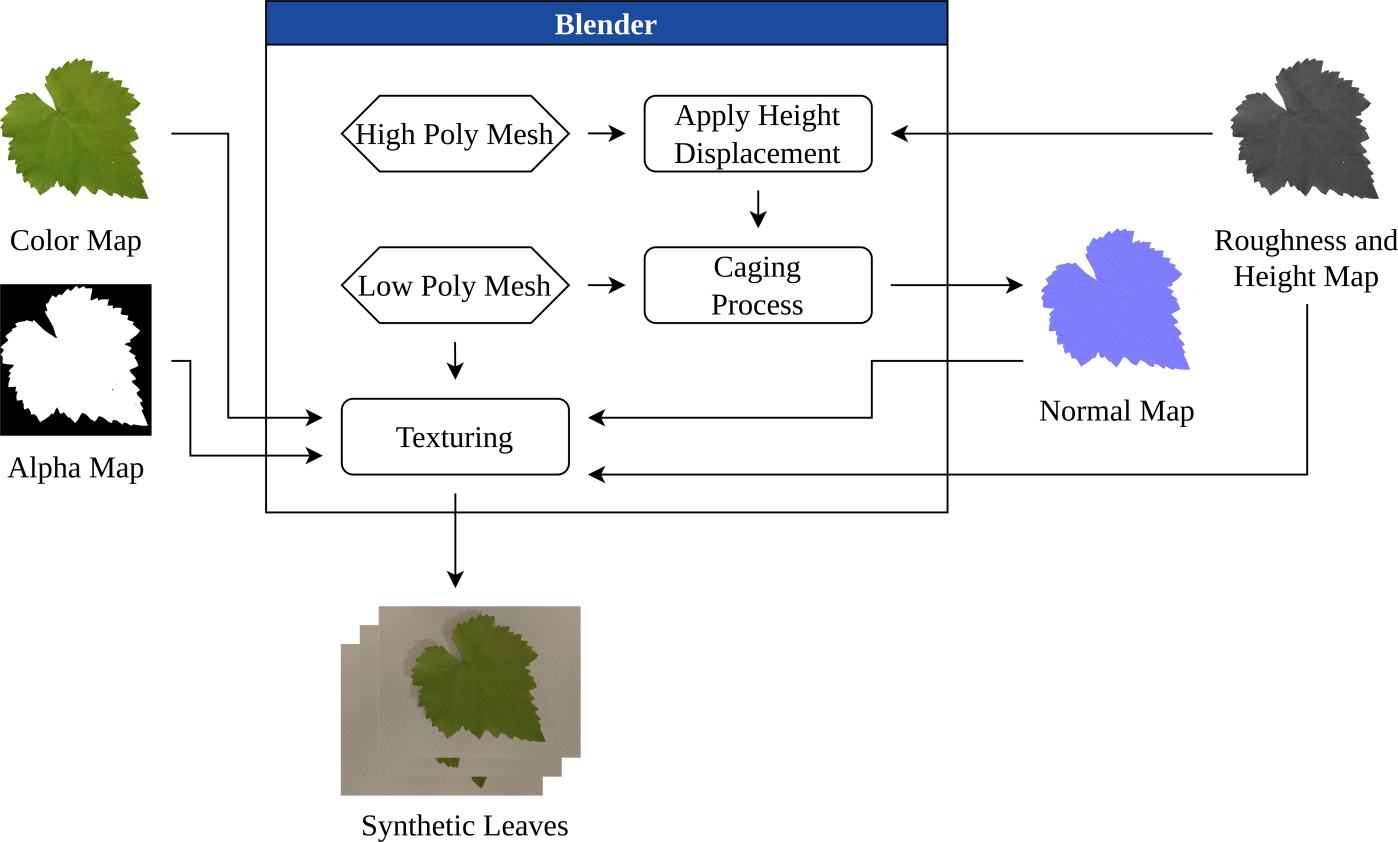
Texture combination stage diagram.

Finally, combining each of these maps, the material T was constructed to form realistic base textures, which could then be systematically varied for further stages of the generation pipeline. This stage of the process is presented in **Figure 3**.

The resulting textures provided a controllable foundation for the subsequent application of disease symptoms and environmental variation.

#### 3.1.2 Disease pattern modeling

Building on the base material T, VitiForge introduces disease symptoms as stochastic perturbations D applied over the leaf surface S. Those perturbations are defined as


$$D=(m:S\to \{0,1\},\;\delta_{a}:S\to {\left[0,1\right]}^{3},\;\delta_{\rho }:S\to [0,1],\;\delta_{n}:S\to S^{2}),$$

where *m* is a binary infection mask indicating symptomatic regions, $\delta_{a}$ chromatic perturbations to the albedo map, $\delta_{\rho }$ perturbations to the roughness field, and $\delta_{n}$ perturbations to the normal field. The resulting diseased material is obtained through


$$T^{\prime }(T,D)=(\text{a}+\delta_{a},\;\alpha ,\;{\text{n}}_{t}+\delta_{n},\;\rho +\delta_{\rho })⊙m,$$

with ⊙ denoting masking, ensuring that modifications are applied only to symptomatic regions.

The disease textures for Black Rot, Leaf Blight, and Esca were generated using the node-based material system in Blender, which allows the combination of texture masks within this formalism through mathematical operations (Guerrero et al., 2022). This approach enables flexible and non-destructive editing: by adjusting parameters such as position, scale, and distribution, disease textures can be systematically varied without recreating them from scratch.

Black Rot was simulated using a layered system designed to reproduce its characteristic concentric lesion structure. Four chromatic perturbations $\delta_{a}$ were stacked: a pale yellow halo, a dark brown border, a lighter brown interior, and a light gray core corresponding to advanced infection. The mask m was initialized from a Photoshop-derived base aligned with the leaf’s vein structure, and subsequently distorted with Perlin noise to introduce variability and organic irregularity. A Voronoi-based submask defined lesion boundaries, mapping grayscale intensities to the layered color bands to mimic progression from mild to severe infection. Normal perturbations $\delta_{n}$ and roughness perturbations $\delta_{\rho }$, also derived from Perlin noise, were applied to blend the lesions into the underlying leaf texture through a color ramp.

Esca was modeled to replicate its distinct chromatic progression. Three layers of $\delta_{a}$ were used: a thin greenish outer zone, a broader yellow band, and a central reddish-brown necrotic area. The mask *m* reused the venation-guided base from Black Rot, combined with procedural noise to enhance irregularity. Multiplying both masks produced a grayscale distribution mask, which was further distorted to simulate natural variability and progression. Color mapping was then applied, reproducing the yellow-to-red transition observed in reference samples, with manual adjustments made to capture tonal differences across leaves. Finally, a noise-based normal perturbation $\delta_{n}$ was applied to simulate subtle surface deformation.

Leaf Blight was reproduced as larger, irregular blotches with a characteristic two-tone structure: a thin yellowish border and a dark gray interior interspersed with scattered brown and light gray spots. A Voronoi-based mask was created and randomly distorted to form the coarse small speckles, while a secondary noise mask generated larger blotches. The combined mask m was mapped to the two-tone $\delta_{a}$ pattern. Overlays of noise textures added the small brown and light gray specks, simulating fungal mycelium. As with the other classes, normal perturbations $\delta_{n}$ and roughness shifts $\delta_{\rho }$ were applied to the infected regions, giving the surface a roughened, irregular texture.

**Figure 4:**
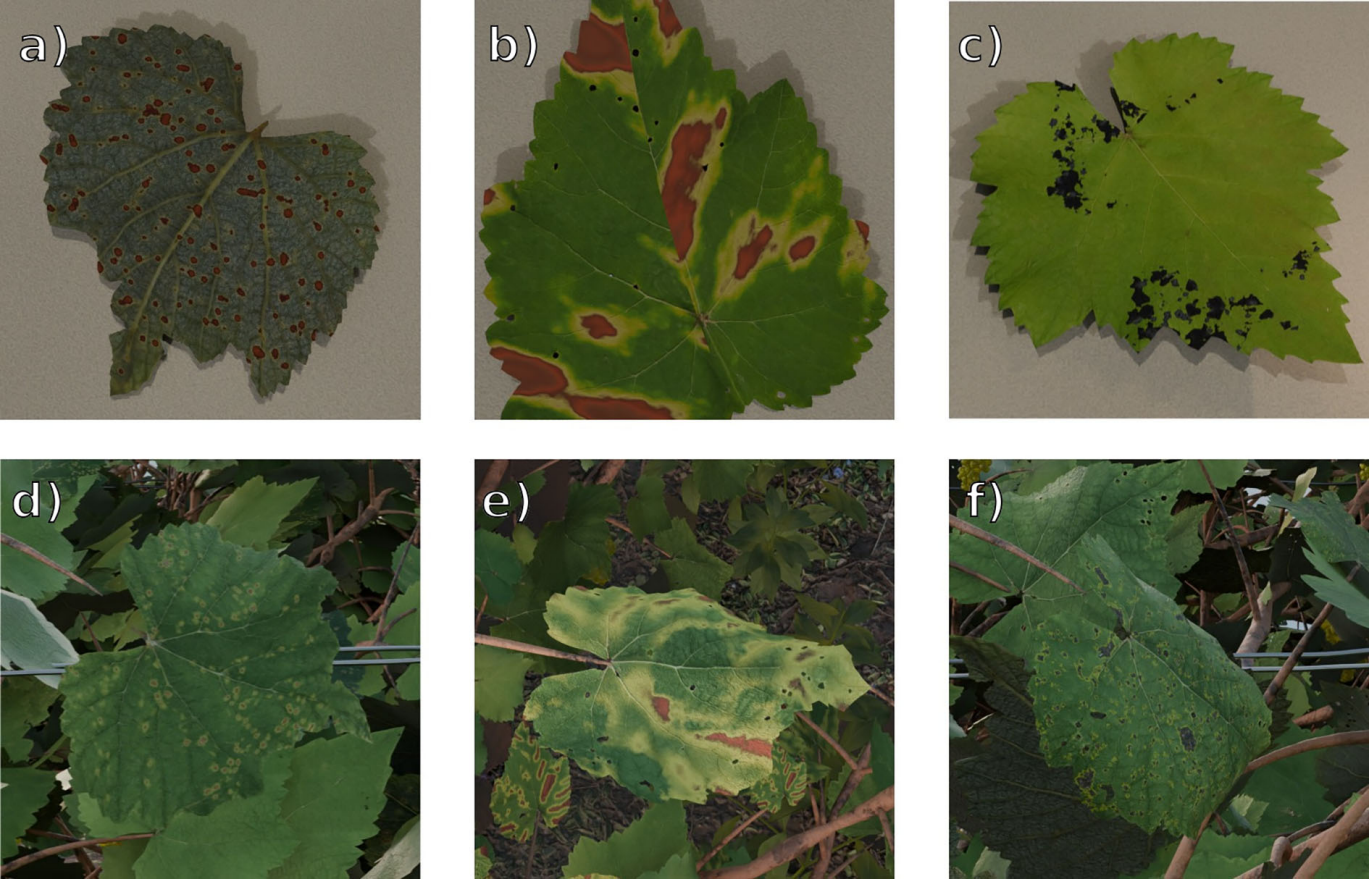
VitiForge diseased leaf generation examples, under “laboratory” and “real-world” rendering conditions. From top to bottom, left to right, **(a)** laboratory Black Rot, **(b)** laboratory Esca, **(c)** laboratory Leaf Blight, **(d)** real-world Black Rot, **(e)** real-world Esca, **(f)** real-world Leaf Blight.

Examples of procedurally generated grapevine leaves for the three disease classes are presented in **Figure 4**, illustrating both controlled and realistic rendering conditions.

#### 3.1.3 Synthetic dataset generation system

**Figure 5:**
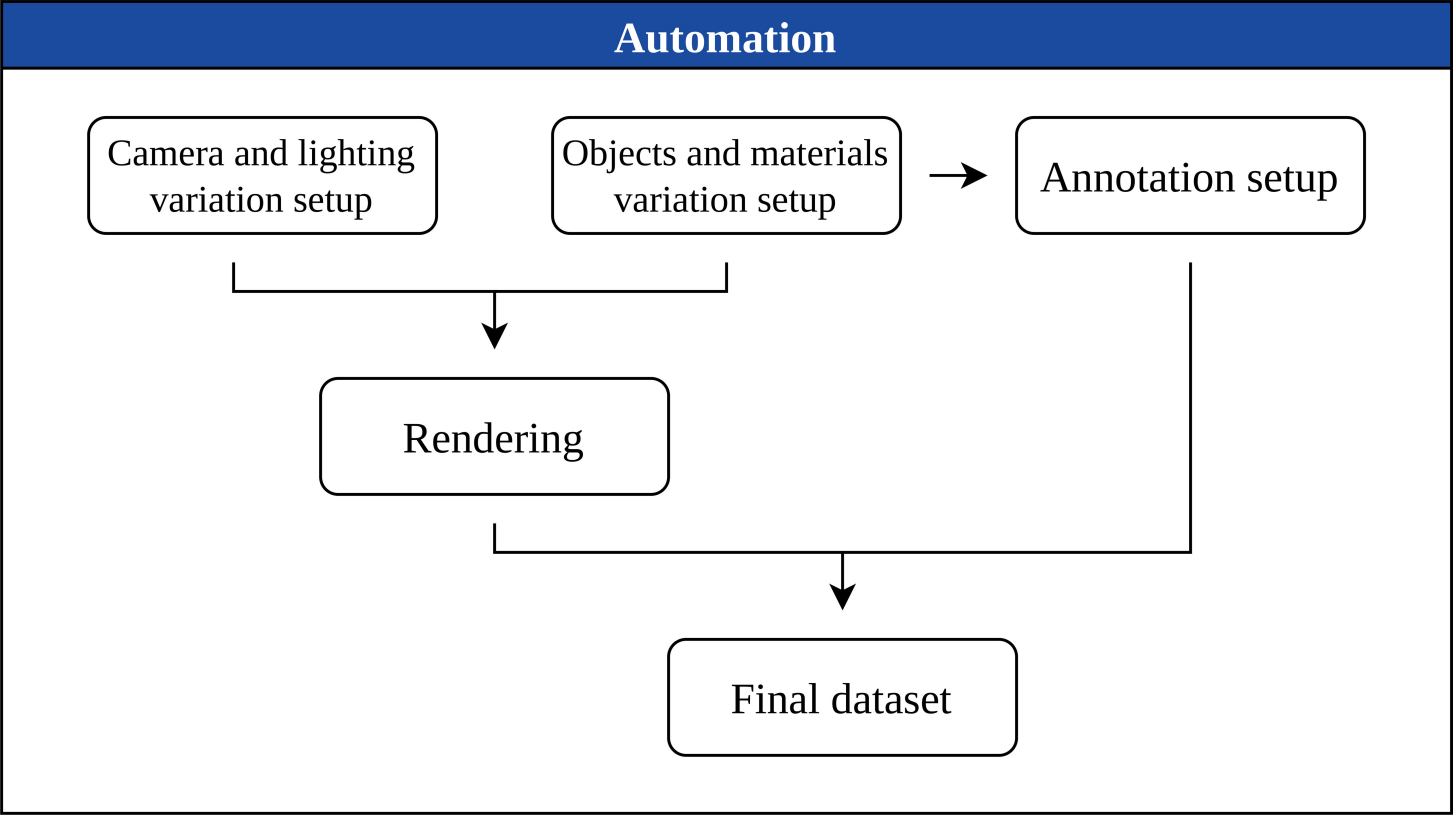
Synthetic dataset generation system.

With the modeling and material definitions established for each class, the dataset generation pipeline was implemented through Blender’s Python API. The system dynamically manipulates material properties, object parameters, and scene configurations to produce a large and visually diverse collection of synthetic images. The full automation workflow is illustrated in **Figure 5**.

The process operates in iterative cycles. In each iteration, a random leaf mesh is selected and assigned one of the pre-defined materials corresponding to the target classes. Parameters controlling disease texture mapping–such as scale and displacement–are randomized within controlled ranges, and deformation modifiers are applied to introduce geometric variability. Leaf orientation is further diversified by rotating the model along all three axes.

Lighting and viewpoint are varied at every cycle. Illumination intensity and color temperature are adjusted within pre-set limits, while light sources and the camera are repositioned within bounded regions and rotated around their own axes, ensuring higher visual diversity.

Each cycle generates two main outputs: a photorealistic render of the leaf and a label file containing the class ID, ensuring compatibility with common machine learning pipelines. The system also produces a semantic segmentation render, providing a foundation for future applications such as bounding box derivation or segmentation-based models. From parameter adjustment to final annotation, the entire cycle executes in approximately 10 seconds, enabling the creation of customizable synthetic datasets.

#### 3.1.4 GAN-based leaf generation

GANs are widely used in agricultural computer vision for data augmentation, particularly when real datasets are limited or imbalanced. Following conventional practice in the plant disease image synthesis literature (Jin et al., 2022; Cap et al., 2022; Li et al., 2024), GAN-based models were adopted here as a baseline strategy against which the proposed procedural pipeline can be evaluated. These models provide a commonly used framework for generating realistic diseased leaf images, especially when paired healthy/diseased datasets are not available. Comparing procedurally generated synthetic leaves with GAN generated images enables an assessment of the relative merits of both approaches in terms of structural fidelity, realism, and utility for downstream classification.

Two GAN architectures were evaluated: CycleGAN (Zhu et al., 2017), a general-purpose unpaired image-to-image translation framework, and LeafGAN (Cap et al., 2022), a plant-specific adaptation that incorporates segmentation maps and shape-aware loss to better guide disease placement. The following subsections outline their comparison and describe the CycleGAN setup used in this study.

#### 3.1.5 Comparison of GAN architectures

CycleGAN (Zhu et al., 2017) employs two pairs of generator–discriminator networks and is trained using a cycle-consistency loss, which ensures that an image translated from domain *A* to *B* and then back again reconstructs the original input. In this study, it was trained to translate healthy grape leaves into diseased counterparts with Black Rot, Esca, and Leaf Blight, while preserving leaf structure and venation.

**Figure 6:**
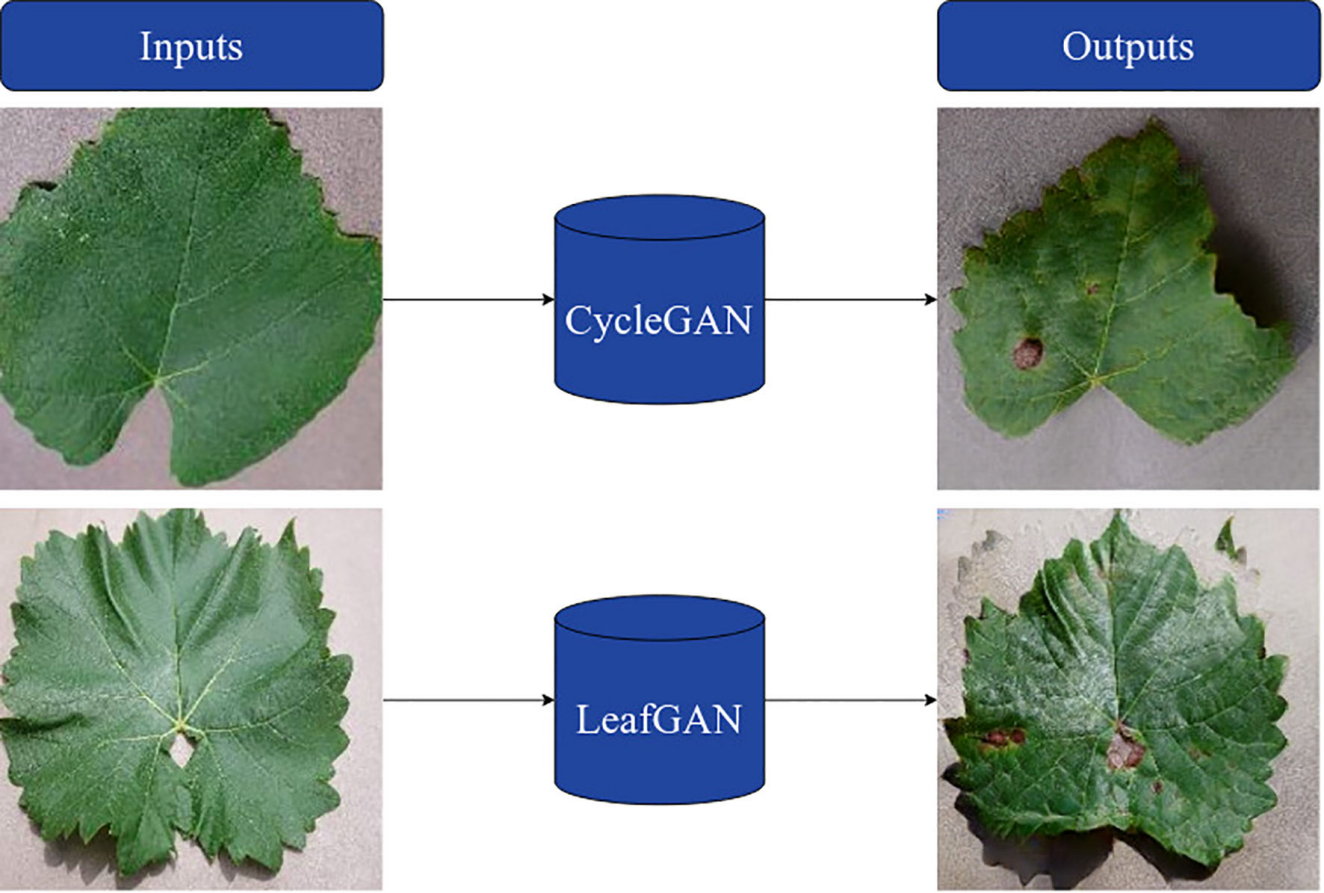
Example outputs of CycleGAN & LeafGAN models.

Proposed by Cap et al. (2022), LeafGAN extends the CycleGAN framework by incorporating segmentation masks and a shape-aware loss function to anatomically constrain disease placement. This design enables lesions to be localized to meaningful regions of the leaf surface, and has shown strong performance in datasets where segmentation masks are reliably available, such as cucumber disease datasets. However, when applied to vineyard data with fewer samples (4,639 compared to 12,000 in cucumber), segmentation guidance proved less stable. Limited annotations often led to irregular disease localization and inconsistent outputs. In comparison, CycleGAN, without relying on segmentation, produced structurally intact leaves and more consistent lesion patterns under the same conditions, as shown in **Figure 6**.

These observations illustrate that segmentation-driven models such as LeafGAN are contingent on dense, high-quality annotations, which were not available for vineyard datasets. CycleGAN, in contrast, provided greater robustness in standardized conditions, synthesizing structurally coherent leaves without requiring segmentation guidance. Nevertheless, in domains with reliable segmentation masks, LeafGAN’s shape-aware constraints may offer advantages by guiding lesion placement with higher anatomical fidelity.

#### 3.1.6 CycleGAN training setup

The CycleGAN models were optimized following the standard training configuration introduced by Zhu et al. (2017), employing adversarial, cycle-consistency, and identity losses alongside the associated hyperparameters and training protocol. Dropout was not used, and training was conducted with a batch size of 1 using the Adam optimizer with an initial learning rate of 0.0002. A linear learning rate decay was applied beginning at epoch 100 to stabilize convergence during later training stages.

Input images were first resized to 266×266 pixels and then cropped to 256×256 pixels, before being normalized to the range [−1,1]. Cycle-consistency and identity losses were weighted at $\lambda_{A}=10.0$, $\lambda_{B}=10.0$, and $\lambda_{\text{identity }=\;}0.5$, respectively.

For each ablation level described in Section 3.2, three separate CycleGAN models were trained, one for each disease class (Black Rot, Esca, and Leaf Blight), using healthy–disease domain pairs. The trained models were then applied to healthy leaves to generate additional diseased samples, which were incorporated into the datasets for the ablation experiments.

### 3.2 Ablation study methodology

To evaluate the effectiveness of synthetic grape leaf images in data augmentation, a data ablation strategy was employed. Models were trained and tested under three experimental cases: (i) real data only, (ii) real data with GAN-based synthetic augmentation, and (iii) real data with VitiForge augmentation. For each condition, progressively greater subsets of real data were used to simulate scenarios of limited dataset availability, enabling the impact of synthetic augmentation on model performance to be systematically assessed.

Although data ablation studies are less common in agricultural computer vision, they are well established in other fields For example, Nowruzi et al. (2019) demonstrated that fine-tuning synthetic data with limited real samples improved detection performance on cars and people using SSD-MobileNet. Likewise, Mousavi et al. (2020) developed an ablation tool and showed that high-fidelity synthetic training can match or even surpass real data in some contexts.

In agriculture, however, the approach is arguably even more pertinent. Collecting annotated field data is both time-consuming and logistically challenging, while certain diseases may be region-specific or restricted to particular growth stages, making them underrepresented in datasets. As a result, agricultural datasets often exhibit scarcity and imbalance, especially for rare or early-stage disease symptoms.

Within this context, the data ablation study explores how much real data is needed to achieve acceptable model performance, whether VitiForge or GAN-based augmentation provides greater benefit when data is limited, and how performance scales as real data becomes more abundant.

#### 3.2.1 Dataset descriptions

For these experiments, five datasets were prepared: one training set, two real testing sets, and two procedurally generated synthetic sets. The real datasets are derived either from the well-known PlantVillage benchmark or from FieldVitis, a curated collection of grape leaf images from the field assembled from multiple public sources. Finally, the synthetic datasets were each generated to mirror the conditions of the corresponding test datasets. All datasets share the same four-class structure: healthy, black rot, esca, and leaf blight, which allows direct comparison across controlled, field, and synthetic conditions.

The training dataset was sourced from Geetharamani and Pandian (2019), which augmented the original PlantVillage dataset (Mohanty et al., 2016). It consists of 950 images and was intentionally left imbalanced, with around 5 times more of healthy leaves than diseased ones, reflecting common collection scenarios where healthy samples are more readily available. This deliberate imbalance creates a more challenging scenario for augmentation techniques to address class imbalance.

**Figure 7:**
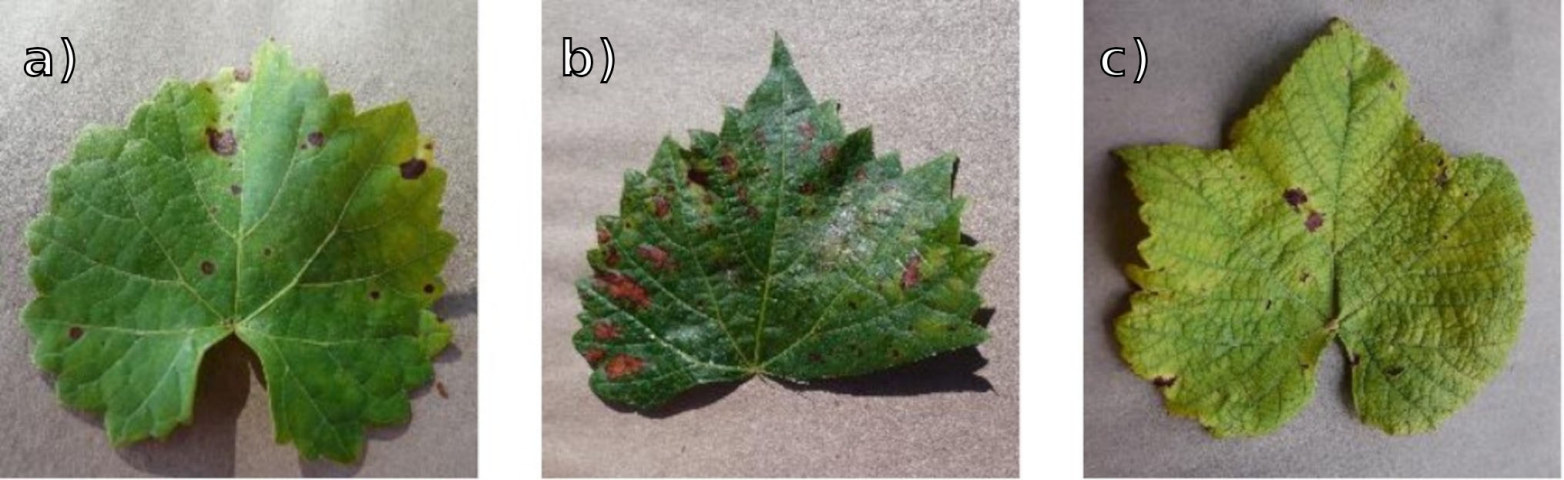
PlantVillage examples. From left to right, **(a)** Black Rot, **(b)** Esca, **(c)** Leaf Blight.

The PlantVillage testing dataset is fully independent of the training set, though also derived from Geetharamani and Pandian (2019). It contains 3,689 images and serves to evaluate performance under laboratory conditions, characterized by uniform lighting, clean backgrounds, and individual leaves with no occlusion. Examples of PlantVillage leaves are shown in **Figure 7**.

**Figure 8:**
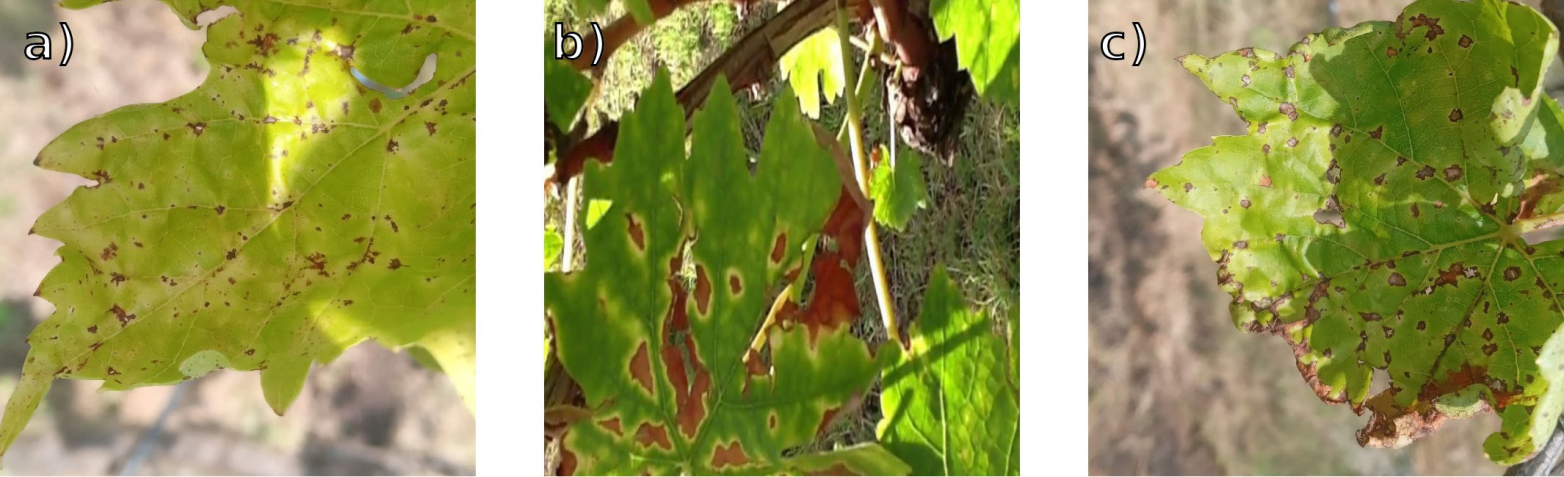
FieldVitis examples. From left to right, **(a)** Black Rot, **(b)** Esca, **(c)** Leaf Blight.

The FieldVitis testing dataset (Farinati Leite et al., 2025) was compiled from several independent public sources. Healthy and Esca images were collected from Alessandrini et al. (2021), Black Rot and Leaf Blight from Shikalgar et al. (2024), with additional Black Rot samples from Singh et al. (2020). Some classes required substantial cleaning: for example, Black Rot contained mislabeled Esca samples, while Leaf Blight included images with disrupted backgrounds such as hands holding leaves. After these revisions, the dataset comprises 555 images and reflects typical field variability, including different lighting conditions (time of day and capture angles), diverse backgrounds (e.g., grass beneath leaves or vineyard canopy), and varied leaf orientations, sometimes even in shadow. Examples are shown in **Figure 8**.

Two VitiForge-based synthetic datasets were created, each designed to approximate the conditions of the test datasets. Synthetic 1 comprises 13,202 samples and corresponds to the PlantVillage test dataset, while Synthetic 2 contains 5,180 samples and corresponds to the FieldVitis test dataset (note that while these sample counts are fixed for the data ablation experiments, VitiForge allows both datasets to be scaled arbitrarily). They were used both to balance and to increase the number of samples during the ablation experiments.

**Table 1** summarizes the distribution of images across the four classes in each dataset.

**Table 1:** Number of images per class across datasets.

Dataset	Healthy	Black rot	Esca	Leaf blight	Total
Training	800	50	50	50	950
PlantVillage	200	1130	1333	1026	3689
FieldVitis	158	39	200	158	555
Synthetic 1	3287	3265	3309	3341	13202
Synthetic 2	1191	1358	1242	1389	5180

#### 3.2.2 Ablation strategy

The ablation strategy was designed to systematically test the impact of synthetic data under different levels of real data availability. By progressively increasing the size of the training dataset, the experiment simulates scenarios where annotated samples are scarce, a common occurrence in this domain. At each reduction step, model performance was evaluated under three training configurations: real data only, real data with VitiForge augmentation, and real data balanced with GAN-generated samples.

The procedure unfolded as follows:

1. Dataset reduction: as described in Section 3.2.1, the original training set contained 950 images, consisting of 800 Healthy leaves and 50 samples per disease class. To simulate different levels of scarcity, progressively bigger subsets were sampled, ranging from 5% to 100% of the original size. For example, at the 10% level, the subset contained approximately 80 Healthy images and 5 images per disease class. Importantly, the natural imbalance between classes was preserved at all levels. These progressively reduced splits serve as the x-axis in the ablation plots.
2. Baseline training: for each subset size, a classification model was trained using only the available real images. These models served as baselines against which augmented training strategies were compared.
3. VitiForge augmentation: each reduced subset was balanced with procedurally generated samples until all classes matched the size of the majority class (e.g., healthy). Beyond this balanced setup, additional synthetic samples were progressively introduced to create different real–synthetic ratios. These experiments were carried out to determine how classification performance varied with increasing proportions of synthetic data, and to identify the optimal ratio for each ablation level based on the classification metrics employed.
4. GAN-based augmentation: for comparison, GANs were trained separately at each reduction level using only the available real images from that subset, ensuring no information leaked from larger datasets. CycleGAN was employed as the primary model. This approach is inspired by prior works that demonstrate the effectiveness of GANs for oversampling imbalanced datasets in computer vision and plant disease diagnosis tasks (Cap et al., 2022; Sampath et al., 2021). The GAN-generated images were then used to balance the reduced subsets, following the same per-class equalization strategy applied in procedural augmentation.
5. Evaluation: models were evaluated using precision, recall, accuracy, and F1-score. Model checkpoints were selected based on the best F1-score. To ensure robustness and reduce variance from random splits, performance was computed using 5-fold cross-validation at each ablation level. For each metric, the mean and standard deviation across folds were calculated and visualized in the line plots, enabling direct comparison of performance trends between the three scenarios.

**Figure 9:**
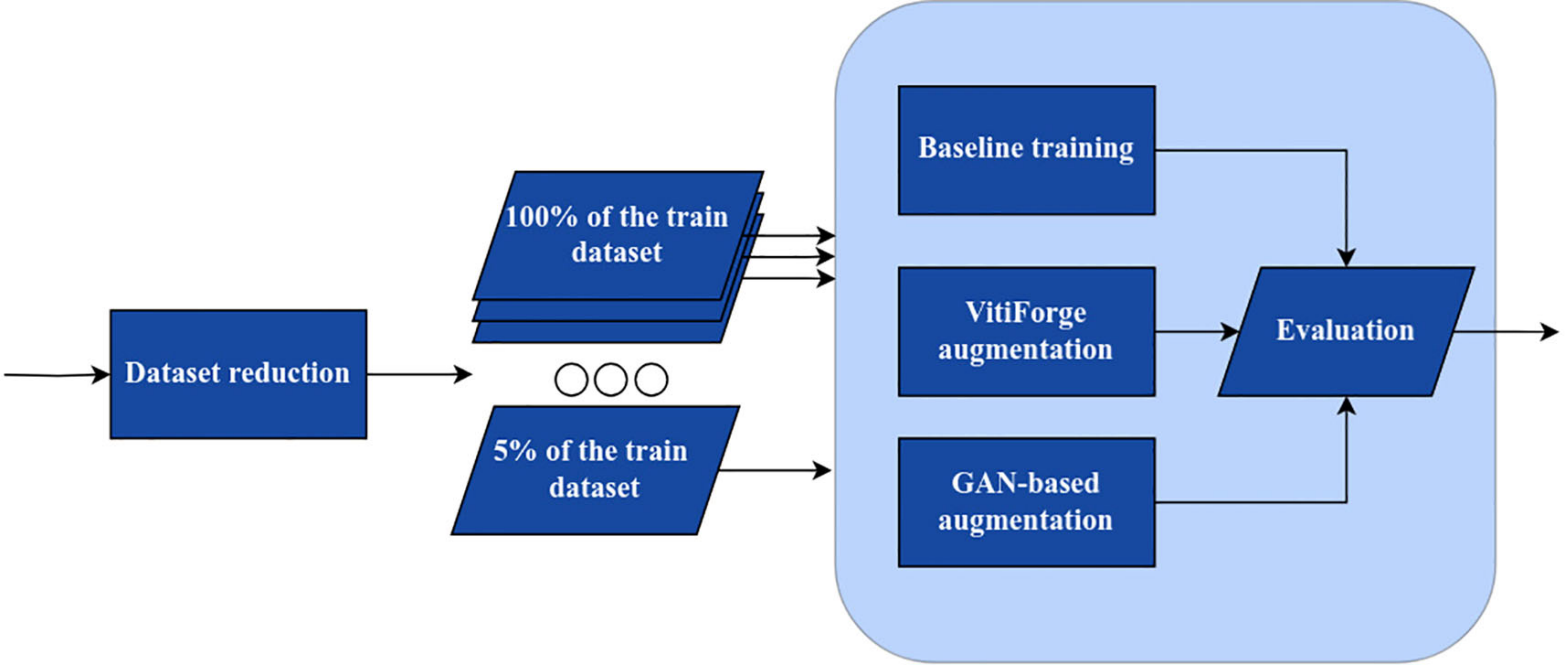
Data ablation pipeline.

**Figure 10:**
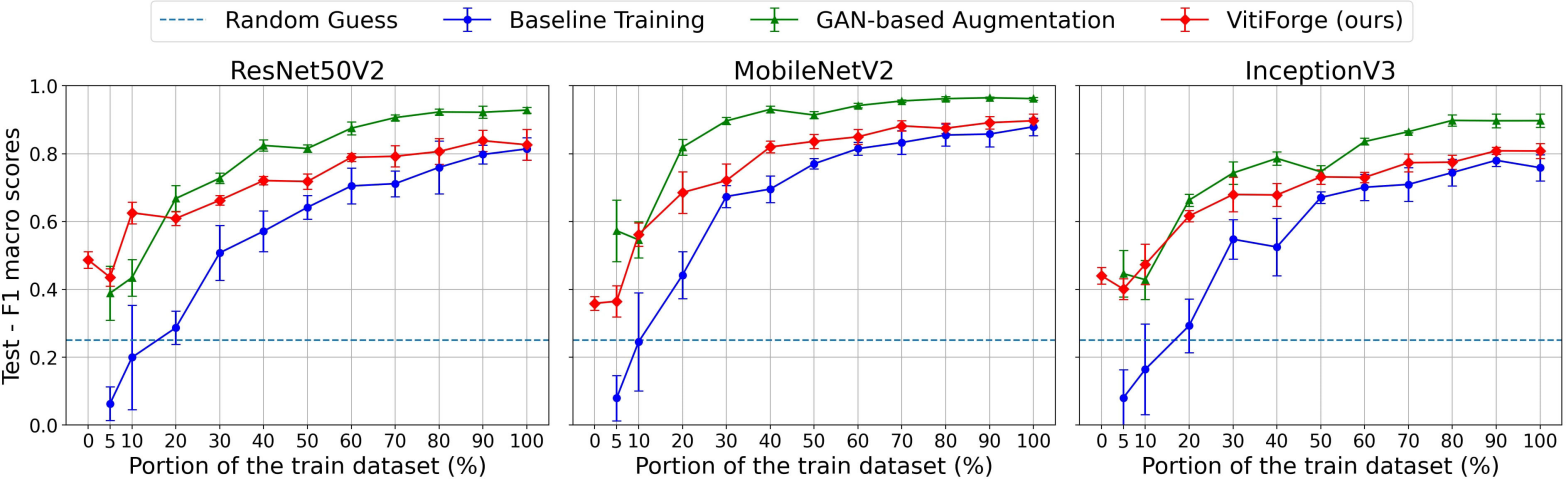
F1 macro scores on the PlantVillage test set across ablation levels.

**Figure 11:**
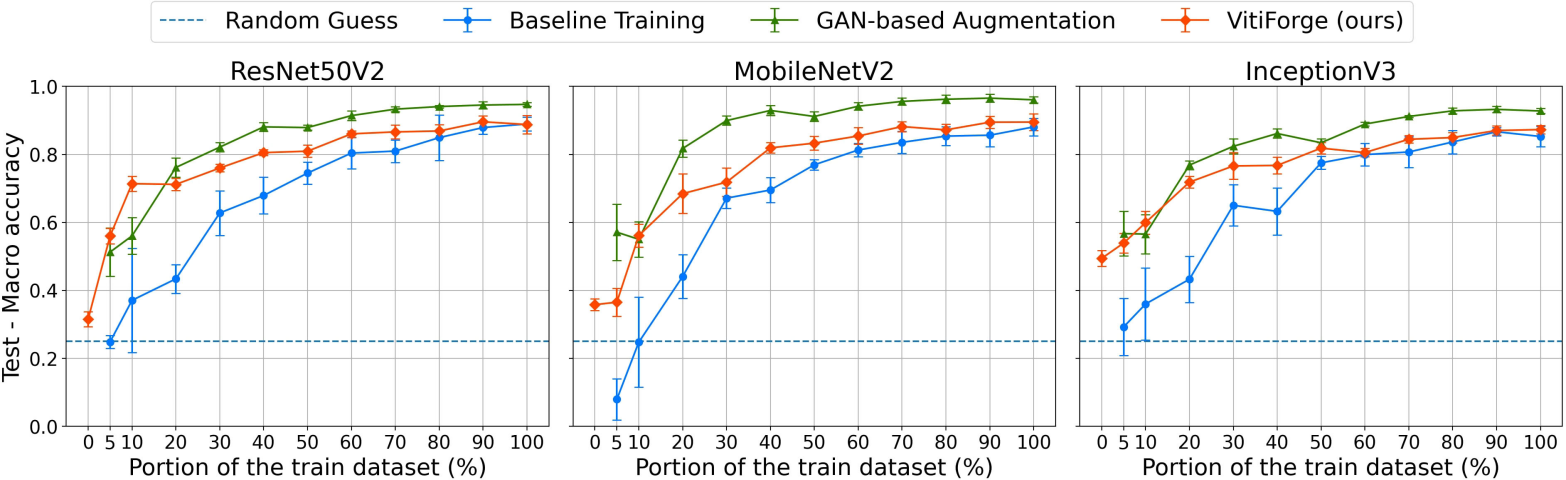
Macro accuracy on the PlantVillage test set across ablation levels.

**Figure 12:**
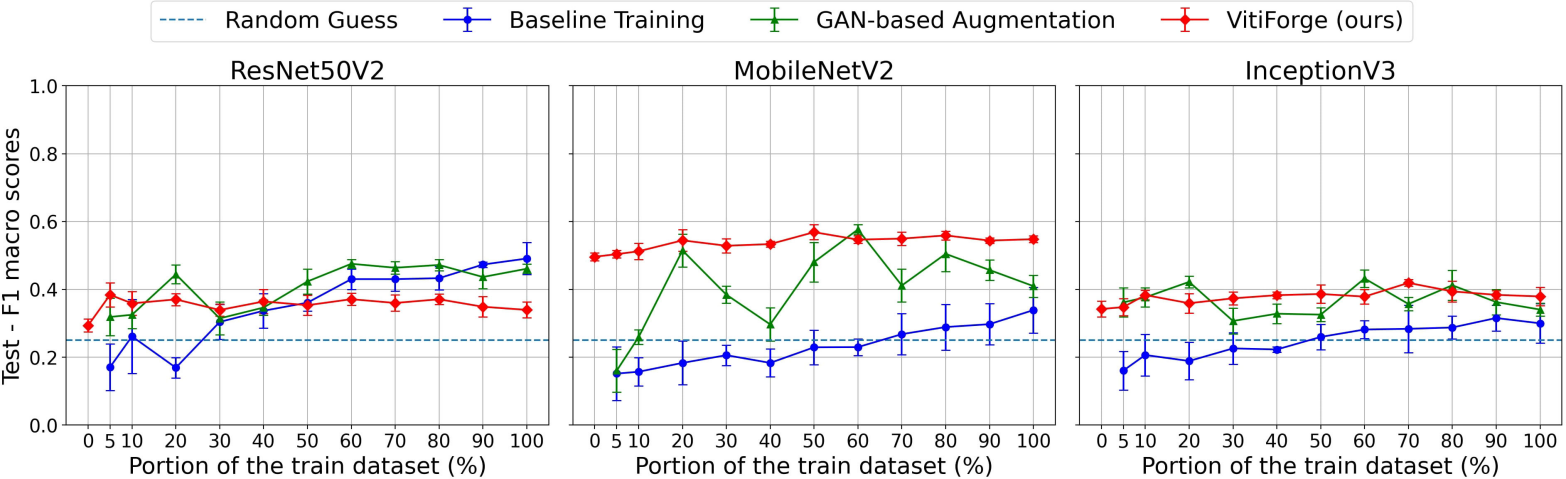
F1 macro scores on the FieldVitis test set across ablation levels.

**Figure 9** illustrates the ablation process, showing how subsets of the training dataset used in the three experimental scenarios.

**Figure 13:**
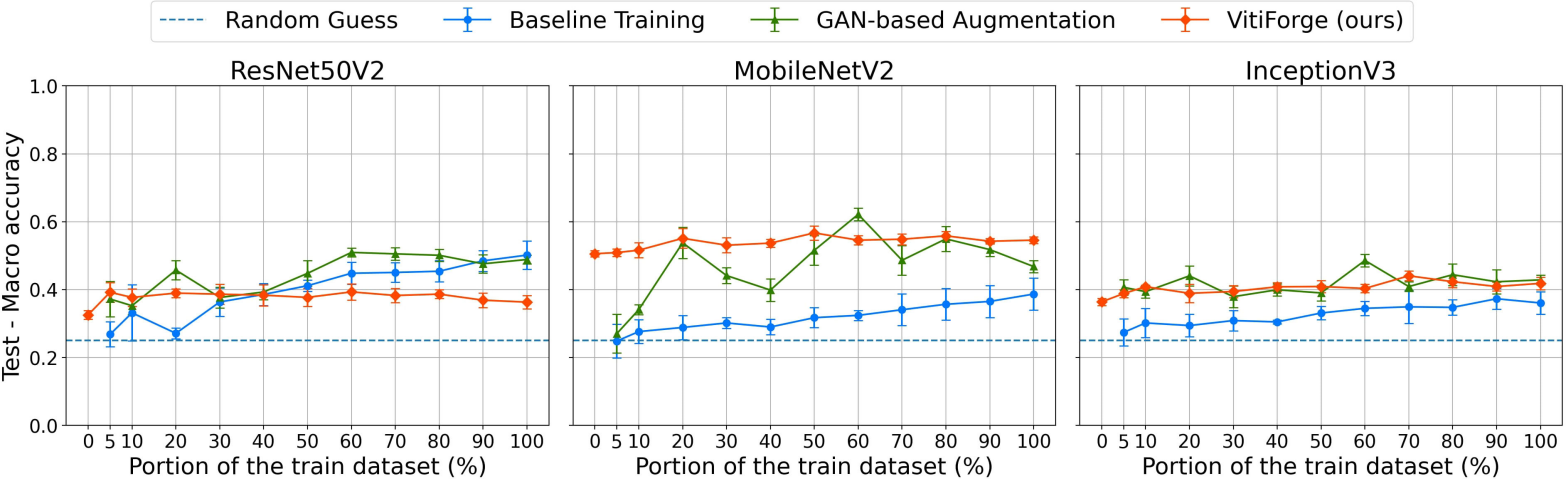
Macro accuracy on the FieldVitis test set across ablation levels.

### 3.3 Model architectures

Three CNN architectures were selected as classifiers for the ablation procedure: MobileNetV2, InceptionV3, and ResNet50V2. These models were chosen because of their computational efficiency, their widespread adoption as benchmarks in image classification, and their demonstrated use in plant disease diagnosis (Karthik et al., 2024a, b; Geetharamani and Pandian, 2019; Fraiwan et al., 2022).

This selection supports reproducibility, as all three models are readily available in major deep learning frameworks. Their efficiency allows experiments to be repeated across multiple ablation levels without prohibitive computational cost. Moreover, their compact design makes them suitable for potential deployment in resource-constrained agricultural settings. A brief overview of each architecture is given below:

- MobileNetV2 (Sandler et al., 2018): designed for mobile and embedded vision applications, it employs inverted residual blocks and depthwise separable convolutions, making it efficient in terms of parameter count and inference speed.
- InceptionV3 (Szegedy et al., 2016): leverages factorized convolutions and dimensionality reduction to extract features at multiple scales, achieving high accuracy with relatively modest computationalrequirements.
- ResNet50V2 (He et al., 2016): introduces residual learning with identity mappings and pre-activation, facilitating the training of deeper models by alleviating vanishing gradient issues.

By employing these three distinct architectures, the study aims to abstract away model-specific differences in performance, allowing the impact to be more confidently attributed to dataset composition and augmentation strategies rather than to particular model designs.

#### 3.3.1 Training setup

All three CNN architectures were initialized with ImageNet-pretrained (Deng et al., 2009) weights and fine-tuned on the augmented training dataset splits, as described in Section 3.2.2. The final classification layers were replaced with a fully connected head producing four outputs corresponding to the target classes (healthy, black rot, esca, leaf blight).

Data preprocessing involved scaling pixel values to the range [-1, 1] and resizing all samples to 224×224 pixels. When optimizing the real–synthetic ratio, random erasing was applied as an additional augmentation technique, using an erasing factor of 0.7 and a scale of 0.08.

Experiments were carried out using TensorFlow 2.19 with CUDA 12.2 and Python 3.11.2 on a Debian 12.11 system equipped with an NVIDIA Quadro RTX 4000 GPU with 8 GB of memory. A batch size of 32 and 10 training epochs were used, the latter empirically chosen to balance performance with the large number of experimental runs required for the ablation study.

Optimization was performed using the Adam optimizer with a learning rate of 0.001 and a weight decay of 0.0002. A dropout rate of 0.3 was applied to reduce overfitting, and the loss function was categorical cross-entropy. Hyperparameters were adapted from Karthik et al. (2024b), which proposed these values after extensive testing on similar architectures.

#### 3.3.2 Evaluation metrics

Model performance was assessed using the F1-score and accuracy, with precision and recall computed as intermediate quantities. The F1-score, defined as the harmonic mean of precision and recall, was chosen to better reflect performance under class imbalance, while accuracy was included as a complementary measure of overall correctness. In addition, loss was monitored to evaluate convergence.

The precision, recall, F1-score, and accuracy for each class were defined as:


$$\text{Precision}=\frac{TP}{TP+FP}$$

$$\text{Recall}=\frac{TP}{TP+FN}$$

$$\text{F}1-\text{Score}=\frac{2\cdot \text{Precision}\cdot \text{Recall}}{\text{Precision}+\text{Recall}}$$

$$\text{Accuracy}=\frac{TP+TN}{TP+TN+FP+FN}$$

where *TP*, *TN*, *FP*, and *FN* denote true positives, true negatives, false positives, and false negatives, respectively.

Each reduced dataset was split into 80% training and 20% validation, and metrics were computed during training on the validation splits, for model checkpointing. Scores were calculated per class and then macro-averaged across the four categories, according to:


$$M_{\text{macro}}=\frac{1}{C}\sum_{i=1}^{C}M_{i}$$

Results across ablation levels (0%–100% of training data) were visualized using scatter plots to compare the three training strategies. The 0% case was also included to assess the feasibility of training exclusively on procedurally generated synthetic data.

## 4 Results

The results of the data ablation experiments on the PlantVillage and FieldVitis test datasets are presented in this section. Models were evaluated across ablation levels ranging from 0% to 100% of the training data under three strategies: real data only, real data balanced and augmented with VitiForge, and real data balanced with GAN-generated samples. Performance was measured using the F1 macro score and macro accuracy, computed with 5-fold cross-validation, and the mean and standard deviation across folds were visualized with scatter plots to assess performance trends under varying levels of data availability.

Results are reported separately for the PlantVillage test dataset, which represents controlled laboratory conditions, and the FieldVitis test dataset, which represents more variable field conditions.

### 4.1 PlantVillage data ablation

**Figure 10** presents the F1 macro scores, on the PlantVillage test set, obtained across ablation levels for MobileNetV2, InceptionV3, and ResNet50V2. **Figure 11** shows the corresponding macro accuracy results under the same conditions.

Across all three architectures, performance increased steadily as more real data became available. At low ablation levels (below 20%), the real-only models exhibited the lowest scores, reflecting the scarcity and imbalance of the available training data. Augmentation improved performance in these regimes, with VitiForge providing early gains across all architectures. This effect was most evident in MobileNetV2, where procedural augmentation consistently produced a small edge at the lowest data proportions. InceptionV3 showed the same trend, although the advantage was less pronounced, while ResNet50V2 also benefited from procedural data at the smallest splits.

At intermediate levels (20%–50%), the gap between augmented and non-augmented training narrowed and the differences between the two augmentation strategies became clearer. In all three architectures, GAN-based augmentation began to surpass procedural synthesis from the 20% split onwards. The effect was especially marked in MobileNetV2, which recorded a noticeable spike with GAN-generated data at this point. InceptionV3 and ResNet50V2 followed a similar trajectory, showing procedural gains in the low-data regime but stronger results from GANs as more real data was introduced.

At higher ablation levels (above 50%), differences among the three strategies became less pronounced. All models approached their maximum performance when trained on the full dataset, though both augmentation strategies continued to outperform real-only training. In this regime, GAN-augmented models generally achieved slightly stronger results than VitiForge.

The accuracy curves closely mirrored the F1 trends, confirming that the observed improvements were not confined to class-balance effects. Across ablation levels, both augmentation strategies steadily improved accuracy, yielding, on average, higher values than the corresponding F1 scores.

In the 0% case, VitiForge enabled model training without any real data, achieving approximately 0.4 for both F1 macro and macro accuracy on the PlantVillage test set.

Among the three architectures, MobileNetV2 achieved the overall best results across the ablation study, consistently outperforming InceptionV3 and ResNet50V2 in both real-only and augmented conditions.

### 4.2 FieldVitis data ablation

**Figures 12**, **13** show the F1 macro scores and macro accuracy on the FieldVitis test set for MobileNetV2, InceptionV3, and ResNet50V2 across all ablation levels.

Overall, scores on FieldVitis were consistently lower than those obtained on PlantVillage, reflecting the added complexity of field imagery, characterized by variable lighting, occlusions, and heterogeneous backgrounds. The two metrics followed remarkably similar trends across all ablation levels, suggesting that class imbalance also did not substantially affect the models’ relative performance. Notably, models trained with GAN-based augmentation and real-only data exhibited visibly larger error bars, indicating higher variability across cross-validation folds and sensitivity to the training-validation split.

In the smallest subsets (below 20%), models trained only on real data performed poorly, while augmentation provided measurable improvements. MobileNetV2 consistently benefited from procedural augmentation in all proportions of real data, showing stable gains over real-only training. The best overall result on FieldVitis was obtained at the 50% split, where MobileNetV2 with VitiForge reached an F1 macro score and a macro accuracy of roughly 0.57.

InceptionV3 also benefited from augmentation techniques, particularly at the lowest ablation levels, though the improvements were less pronounced than those observed with MobileNetV2. Both augmentation strategies produced competitive results across the different proportions of real data, and on average, VitiForge maintained a slight advantage.

For ResNet50V2, GAN augmentation consistently performed marginally better than the procedural augmentation. Augmentation overall was less effective for this architecture, with models trained solely on real data producing relatively stronger results compared to the augmented cases at higher ablation levels.

As the amount of real data increased beyond 50%, real-only training showed clear upward trends, while augmented strategies produced more mixed results, occasionally plateauing or yielding inconsistent gains. Nonetheless, both augmentation methods outperformed real-only training at nearly all ablation levels, with VitiForge proving particularly effective for MobileNetV2 in the low-data regime.

At the 0% level, models trained exclusively with procedurally generated data achieved F1 macro scores ranging from 0.35 to 0.5 and corresponding macro accuracies in a similar range across the three architectures.

## 5 Discussion

The ablation experiments from Section 4 highlight distinct roles for procedural and GAN-based augmentation across data regimes and test conditions. Both methods consistently improved performance over real-only training, but their relative strengths varied depending on the availability of real data and the evaluation dataset. VitiForge proved especially valuable in low-data scenarios, where it provided reliable improvements and even enabled training without any real samples. GAN augmentation depended on reaching a minimum dataset size; beyond this threshold, it consistently yielded stronger results provided the gap between the training distribution and the target application was small.

In the PlantVillage dataset, VitiForge excelled in the lowest data scenarios, where its consistent outputs supplied additional examples that supported classifier convergence under scarce real data. Once the proportion of real data exceeded 10%, GAN augmentation began to gain the upper hand, leveraging more complete distributions to capture finer-grained textures and patterns.

In contrast, results on the FieldVitis dataset showed a stronger role for VitiForge. Here, the procedural methodology often matched or surpassed GAN-based synthesis, particularly with the MobileNetV2 architecture. This outcome likely reflects the explicit variability controls embedded in VitiForge (e.g., randomized lighting, orientation, and background noise), which aligned more closely with the diversity of real field conditions than GANs trained on curated PlantVillage data. In particular, classifiers trained exclusively on procedurally generated images achieved competitive scores, demonstrating the potential of VitiForge as a standalone training resource.

GAN-based augmentation remained competitive in higher-data regimes across both datasets, consistently performing well when real training datasets grow larger, allowing them to better capture disease-specific textures. However, its reliance on representative, annotated datasets constrains applicability in field settings, where rare diseases and early infection stages are often underrepresented (Sharma et al., 2024). Furthermore, adversarial training can bias models toward dominant patterns, reducing diversity and limiting coverage of atypical or early-stage disease manifestations. These observations suggest that GANs are best suited as complementary tools for high-fidelity augmentation when data availability is not a limiting factor, while procedural synthesis offers greater adaptability and scalability in cross-domain and low-data scenarios. In contexts requiring broader variability or resilience to annotation scarcity, hybrid or alternative generative methods may be preferable (Muhammad et al., 2023; Müller-Franzes et al., 2023).

Beyond augmentation methods, the choice of model architecture further influenced outcomes, with clear trends emerging across MobileNetV2, InceptionV3, and ResNet50V2. MobileNetV2 consistently achieved the strongest results in this study, aligning with multiple reports in the literature that highlight its effectiveness for grape leaf classification (Thomkaew, 2025; Mangaoang, 2025). InceptionV3 provided a strong and stable baseline but generally fell slightly behind MobileNetV2, while ResNet50V2 occasionally benefited more from real-only training, reflecting its sensitivity to augmentation regimes. Importantly, classification tasks on curated datasets such as PlantVillage are close to “solved”, with near-ceiling accuracies across architectures. As Shah et al. (2023) notes, relatively small differences in preprocessing, augmentation, class balance, or checkpointing criteria can shift performance rankings between the architectures.

## 6 Conclusion

This work investigated the role of synthetic data generation for grape leaf disease classification, comparing VitiForge, GAN-based synthesis, and no augmentation through a systematic data ablation study. Results showed that GAN augmentation excelled on PlantVillage when sufficient real data was available, while VitiForge was more effective in the low-data regime. In the FieldVitis dataset, the procedural rendering technique consistently matched or outperformed GAN augmentation, highlighting its strength in bridging the gap between controlled laboratory datasets and real-world conditions.

Procedural-based synthesis offers key advantages: it eliminates the need for immediate in-field data collection, enabling preemptive training before outbreaks occur. By parameterizing lesion attributes, such as size, number, color intensity, spatial distribution, and blending with leaf venation, along with environmental factors like lighting and background clutter, it produces diverse, balanced, and fully annotated datasets without manual labeling costs. This controlled process allows the simulation of rare, early-stage, or geographically constrained disease cases, which are often absent in real datasets.

GAN-generated images, in contrast, function best as augmentation tools when domain-specific real data is already available. Since GANs require training examples, they cannot be deployed in a zero-data scenario. However, once trained, they can inject realistic texture variations, noise patterns, and morphological diversity, helping improve robustness and generalization across intra-domain variations.

When interpreting these results, some limitations should be acknowledged. First, the experiments were restricted to a limited set of grape diseases, which may not generalize to other pathogens or crop species with more complex visual symptoms. Second, grape cultivar was not a controlled factor in our experiments, and variety-specific differences in leaf morphology may influence recognition outcomes. Third, while VitiForge offers explicit control over lesion characteristics and environmental conditions, it may still fall short of capturing the full biological variability of disease progression observed in real vineyards, especially in mixed infections or under extreme environmental stress. Fourth, GAN-based augmentation was evaluated using a limited set of architectures and training conditions; alternative generative models may yield different outcomes. Finally, the evaluation focused primarily on classification tasks; extending the analysis to segmentation or detection scenarios could reveal additional challenges in domain transfer.

In summary, this paper highlights the complementary roles of procedural and GAN-based synthesis: VitiForge offers orderly control and flexibility during early-stage release, while GANs add realism and diversity during late-stage, when real-world data exists. Looking ahead, future work should explore hybrid frameworks that integrate the controllability of procedural methods with the fidelity of generative models, while extending experiments to additional grapevine pathogens, diverse cultivation contexts (across locations and cultivars), and exploring semantic segmentation outputs for mixed infection recognition. Preprocessing strategies may also help mitigate domain shifts and facilitate the transfer of models trained on laboratory datasets to field conditions, as in Li et al. (2023), thereby complementing the benefits of synthetic augmentation. Finally, integrating these synthetic pipelines into real-time decision-support tools for growers could help advance early disease diagnosis from a research-focused approach into a practical instrument for sustainable viticulture.

## Data Availability

The raw data supporting the conclusions of this article will be made available by the authors, without undue reservation.
